# Integrated Transcriptomic and Metabolomic Analyses Identify Candidate Transcription Factors Associated with Flavonoid and Coumarin Accumulation in *Psoralea corylifolia*

**DOI:** 10.3390/biology15171567

**Published:** 2026-09-07

**Authors:** Zhangyiyi You, Hanhong Liang, Huiting Liao, E Ou, Hongqiu Zhou, Xuanxuan Cheng, Hanjing Yan, Hongyang Gao, Zhong Li

**Affiliations:** 1 School of Traditional Chinese Medicine, Guangdong Pharmaceutical University, Guangzhou 510006, China; yzyyde@163.com (Z.Y.); 2112450127@stu.gdpu.edu.cn (H.L.); 2112348071@stu.gdpu.edu.cn (H.L.); gdyxycxx@126.com (X.C.); lsyhj@gdpu.edu.cn (H.Y.); 2 Guangdong Leiyunshang Pharmaceutical Co., Ltd., Yunfu 527300, China; oue@gd-lys.cn (E.O.); zhouhongqiu@gd-lys.cn (H.Z.)

**Keywords:** *Psoralea corylifolia* L., transcriptome, metabolome, flavonoids, coumarins, tissue specificity

## Abstract

Background: *Psoralea corylifolia* is a widely used traditional medicinal plant, with its dried mature fruits as the main medicinal part. Flavonoids and coumarins are the primary bioactive compounds of this species. However, the tissue-specific metabolic profiles, gene expression patterns, and potential regulatory factors underlying active compound biosynthesis remain largely uncharacterized across different tissues of *P. corylifolia*; Methods: In this study, five tissue types (roots, stems, leaves, flowers and fruits) of *P. corylifolia* were collected as experimental materials. We performed integrated widely targeted metabolomic and transcriptomic analysis, combined with weighted gene co-expression network analysis (WGCNA), to screen co-expression modules and candidate regulatory factors associated with flavonoid and coumarin accumulation; Results: Distinct tissue specificity was observed at both metabolomic and transcriptomic levels among different tissues, with the most remarkable difference between fruits and other tissues. Signature bioactive compounds including isobavachalcone, bavachin and corylin were specifically and highly accumulated in fruits. Differentially expressed genes were mainly enriched in phenylpropanoid biosynthesis, flavonoid biosynthesis and isoflavonoid biosynthesis pathways. WGCNA revealed that the magenta module was significantly positively correlated with fruit tissues and the contents of the above bioactive metabolites. Six candidate transcription factors were identified from this module and classified into three candidate-priority tiers based on a TF–pathway gene co-expression network (344 edges, |r| ≥ 0.8, *p* < 0.05) and connectivity metrics. The prioritized hubs were Cluster_22013.0 (C3H-type zinc finger transcription factor) and Cluster_21217.8 (NF-YA; *Arabidopsis* homolog NFYA9/AT3G20910), with Cluster_9299.0 (Rcd1-like) and Cluster_20910.0 (NAC; *Arabidopsis* homolog NAC002/AT5G04410) as highly connected positively correlated candidates, while Cluster_31326.1 (bZIP) and Cluster_11436.0 (C2H2) were identified as negatively correlated candidates, with all their significant edges representing negative correlations with pathway genes; Conclusions: This study characterizes tissue-specific metabolic and transcriptomic patterns in *P. corylifolia*, and identifies candidate co-expression modules and transcription factors associated with flavonoid and coumarin accumulation in fruits. The prioritized TF tiers, including candidate hub and negatively correlated TFs, provide a foundation for future functional studies on the regulation of active compound biosynthesis in *P. corylifolia*.

## Simple Summary

*Psoralea corylifolia* is a traditional medicinal plant whose dried fruits are widely used to treat skin diseases, osteoporosis, and other conditions. Flavonoids and coumarins are important bioactive compounds in this species, but the basis of their tissue-specific accumulation is not well understood. In this study, we examined metabolite profiles and gene expression in five plant tissues (root, stem, leaf, flower, and fruit) using metabolomic and transcriptomic analyses. Each tissue showed a distinct metabolite and gene-expression profile, and fruit samples formed a distinct cluster clearly separated from the other tissue groups in the PCA plot. By grouping genes with similar expression patterns, we identified candidate gene modules and transcription factors associated with the accumulation of these compounds in fruit. This work is an exploratory screening study, and the identified candidate transcription factors require further experimental validation. Our results provide a valuable genetic and chemical data resource that may serve as a foundation for future research on the biosynthesis of active compounds in *P. corylifolia*.

## 1. Introduction

*Psoralea corylifolia* L. (*P. corylifolia*), an annual erect herb of the legume family (Fabaceae), is widely distributed in China, India, and other Asian regions [1]. As a traditional medicinal plant, its dried ripe fruit is the official medicinal part and has a long history of use in both traditional Chinese medicine and the Indian Ayurvedic system [2,3]. Modern pharmacological studies have confirmed that *P. corylifolia* possesses various biological activities, including estrogen-like [4], antibacterial, anti-inflammatory and antioxidant [5]. It can be used for the auxiliary prevention and treatment of postmenopausal osteoporosis [6], the management of diabetes and obesity [7], the clinical adjunctive treatment of skin diseases such as vitiligo [8], and the alleviation of urinary symptoms such as nocturia and frequent urination [9].

The pharmacological activities of *P. corylifolia* are mainly attributed to its abundant secondary metabolites, among which coumarins and flavonoids are the two most characteristic classes of active constituents [9]. Representative coumarins include psoralen and isopsoralen, whereas characteristic flavonoids include isobavachalcone, bavachin, and corylin [10,11]. It is noteworthy that furanocoumarins such as psoralen and isopsoralen are not only pharmacologically active but have also been reported to pose potential hepatotoxic risks [12]. Therefore, characterizing the accumulation patterns and candidate biosynthetic associations of these active components is important for both enhancing the efficacy and ensuring the safety of *P. corylifolia* as a medicinal material.

Existing studies have shown that both coumarins and flavonoids originate from the phenylpropanoid pathway and share p-coumaroyl-CoA as a common precursor [13,14]. After biosynthesis by phenylalanine ammonia-lyase (PAL), cinnamate 4-hydroxylase (C4H), and 4-coumarate:CoA ligase (4CL), the pathway diverges: the coumarin branch ultimately yields linear and angular furanocoumarins such as psoralen and isopsoralen [15,16]; the flavonoid branch leads to the formation of naringenin chalcone and subsequent flavones, isoflavones, flavanones and flavonols, catalyzed by enzymes such as chalcone synthase (CHS) and chalcone isomerase (CHI) [17,18,19]. Dozens of coumarins and flavonoids have been detected in *P. corylifolia* [20]; however, the genes catalyzing the characteristic skeletal modifications of furanocoumarins, as well as the regulatory factors controlling the tissue-specific expression of these genes, remain largely unreported.

In leguminous plants, important progress has been made in understanding the transcriptional regulation of tissue-specific flavonoid accumulation. For example, MYB40 in *Medicago truncatula* specifically activates flavonoid branch genes in roots and nodules [21], while GmMYB58 and GmMYB205 in soybean (*Glycine max*) act as seed-specific regulators that directionally upregulate the expression of key genes in the isoflavonoid branch [22]. For coumarins, the root-specific biosynthesis of scopolin and other coumarins in *Arabidopsis thaliana* has been demonstrated to be regulated by the transcription factor MYB72 [23]; however, in the genus Psoralea and even in legumes, transcription factors that regulate the tissue-specific accumulation of furanocoumarins have not yet been isolated. Comparative transcriptomic studies within Psoralea are virtually absent, and the lack of gene–metabolite association networks limits the identification of candidate genes associated with tissue-specific differences in active-component accumulation.

With the development of high-throughput sequencing technologies, integrated transcriptomic and metabolomic analyses have become an effective strategy for exploring plant secondary metabolism and screening candidate biosynthetic genes. This approach has been successfully applied in various medicinal plants to identify candidate genes related to the accumulation of active components [24,25]. In this study, five tissues (root, stem, leaf, flower, and fruit) of *P. corylifolia* were used to integrate broadly targeted metabolome and transcriptome sequencing data, combined with weighted gene co-expression network analysis (WGCNA), to identify co-expression modules and candidate transcription factors associated with flavonoid and coumarin accumulation in the fruit. The results provide candidate gene resources and a data foundation for future functional studies on the biosynthetic pathways and regulatory networks of active components in *P. corylifolia*.

## 2. Materials and Methods

### 2.1. Tissue Sampling

*P. corylifolia* plant materials were collected from Plot No. 60 of the Industrial Transfer Park, Duyang Town, Yun’an District, Yunfu City, Guangdong Province, China (geographic coordinates: 23.0224° N, 112.1716° E). The experimental site has an annual average temperature of 22.8–23.0 °C and loose, slightly acidic improved laterite soil. Free from industrial contamination, the area provides suitable ecological conditions for *P. corylifolia* cultivation. All experimental plants were uniformly cultivated one-year-old seedlings of the same age, and all were at the mature growth stage. Sampling was conducted on the morning of 20 November 2025, at which time all plant parts, including fruits, were fully mature. The plant samples were divided into five tissue types: roots, stems, leaves, flowers, and fruits. For each tissue type, three biological replicates were prepared, with each replicate consisting of pooled tissue from six individual plants. All five tissue types shared the same three plant replicate pools, forming a blocked paired sampling design; a total of 18 individual plants were used in this study. Immediately after collection, the samples were rinsed with running water, blotted dry with absorbent paper, flash-frozen in liquid nitrogen, and stored at −80 °C until further analysis.

### 2.2. Reagents and Instruments

All chemical reagents used in this study were of chromatographic grade. Detailed information on the reagents and reference standards is listed in Table S1. The main instruments and equipment used for sample preparation and instrumental analysis are summarized in Table S2.

### 2.3. Widely Targeted Metabolomics Analysis

#### 2.3.1. Sample Preparation

Biological samples were vacuum freeze-dried and ground into fine powder. Briefly, 30 mg of powder was extracted with 1500 μL of 70% methanol aqueous solution (pre-cooled at −20 °C) containing 2-chlorophenylalanine as the internal standard. After vortex-assisted extraction and centrifugation at 12,000 rpm for 3 min, the supernatant was filtered through a 0.22 μm microporous membrane for subsequent UPLC-MS/MS analysis.

#### 2.3.2. UPLC-MS/MS Analytical Conditions

Chromatographic separation was performed on an ExionLC™ AD ultra-performance liquid chromatography system using an Agilent SB-C18 column (SCIEX, Marlborough, MA, USA) (1.8 μm particle size, 2.1 mm × 100 mm). The mobile phase consisted of ultrapure water with 0.1% formic acid (phase A) and acetonitrile with 0.1% formic acid (phase B). The gradient elution program was set as follows: 5% B at 0 min, a linear increase to 95% B over 9 min followed by a hold until 10 min, then a return to 5% B at 11.10 min and equilibration until 14 min. The flow rate was 0.35 mL/min, the column temperature was controlled at 40 °C, and the injection volume was 2 μL.

Mass spectrometric detection was carried out on a triple quadrupole mass spectrometer (SCIEX, Marlborough, MA, USA) equipped with an electrospray ionization (ESI) source, operated in both positive and negative modes with multiple reaction monitoring (MRM) scan mode. The ESI source temperature was set at 500 °C, with ionspray voltages of 5500 V (positive mode) and −4500 V (negative mode). Detailed MRM transition parameters (declustering potential, collision energy) for representative metabolites are provided in Appendix A. The complete set of Q1, Q3, DP, and CE values for all metabolites is provided in Table S3.

#### 2.3.3. Metabolite Identification and Data Analysis

Metabolite annotation was performed by matching accurate mass, MS/MS fragmentation patterns, isotopic distribution of fragment ions, and retention time against the commercial Metware Database (MWDB, Metware Biotechnology Co., Ltd., Wuhan, China) using an intelligent spectral matching algorithm. No authentic standards were run concurrently with the experimental samples; all metabolite identifications were based on database matching. The identification confidence was classified into three levels: Level 1 (matching score ≥ 0.7 with authentic standards), Level 2 (matching score of 0.5–0.7 with database entries), and Level 3 (consistent Q1, Q3, retention time, declustering potential and collision energy with database entries). Isotope signals and redundant adduct ions (K^+^, Na^+^, NH_4_^+^) were excluded during data processing. 2-Chlorophenylalanine was included as an internal standard to monitor instrument stability and was not used for peak area normalization. Quantification was performed based on relative MRM peak areas.

Missing peak area values for individual metabolites were imputed with 1/5 of the minimum peak area of each metabolite across all samples. Metabolites with a coefficient of variation (CV) < 0.5 in quality control samples were retained for statistical analysis, a criterion supported by published widely targeted metabolomics studies [26]. Differentially accumulated metabolites were screened using a combined univariate-multivariate statistical strategy. For pairwise inter-tissue comparisons, two-sample Student’s *t*-test was applied to calculate raw *p*-values of metabolite abundance differences. Homogeneous variances were observed within each tissue group due to high replicate reproducibility; thus, Welch’s *t*-test was not adopted in this study. Orthogonal partial least squares-discriminant analysis (OPLS-DA) was constructed to calculate the variable importance in projection (VIP) value for each metabolite. All pairwise OPLS-DA models were validated with 200 random permutation tests; full diagnostic parameters (R^2^X, R^2^Y, Q^2^) of all nine tissue comparison groups are summarized in Table S4, and representative permutation validation plots are displayed in Figure S1. Metabolites satisfying VIP > 1, |log_2_ fold change| ≥ 1 and raw *p* < 0.05 were defined as differentially accumulated metabolites (DAMs). Metabolite enrichment analysis was performed using MetaboAnalyst (https://www.metaboanalyst.ca, accessed on 1 March 2026) based on over-representation analysis.

#### 2.3.4. Interpretation of Fold Change Results

In the metabolomic analysis, all pairwise tissue comparisons were performed with fruit as the control (“other tissue vs. Fruit”). A positive log_2_ fold change (upregulation) indicates higher metabolite abundance in other tissues relative to fruit, whereas a negative log_2_ fold change (downregulation) indicates lower abundance in other tissues, suggesting that the metabolite is enriched in fruit.

### 2.4. Transcriptomic Analysis

Total RNA extraction, library construction, and transcriptome sequencing were commissioned to Metware Biotechnology Co., Ltd. (Wuhan, China). Total RNA was isolated from *P. corylifolia* tissues using the CTAB method combined with pBIOZOL reagent. RNA purity and integrity were assessed using a Qubit 4.0 fluorometer (Thermo Fisher Scientific, Waltham, MA, USA) and a Qsep400 high-throughput nucleic acid analyzer (BiOptic Inc., New Taipei City, Taiwan, China). mRNA was enriched from total RNA using Oligo(dT) magnetic beads, and then fragmented into short fragments in fragmentation buffer. First-strand cDNA was synthesized using random hexamer primers, followed by second-strand cDNA synthesis. For the strand-specific library, dUTP was incorporated into the second strand in place of dTTP to achieve strand specificity, as the high-fidelity DNA polymerase used in subsequent PCR cannot amplify uracil-containing templates. After end repair and A-tailing, target-size cDNA fragments (250–350 bp) were selected with AMPure XP beads and amplified by PCR to construct strand-specific mRNA libraries. Library quality was verified by Qubit 2.0 quantification, Agilent 2100 insert size detection, and qPCR quantification. Sequencing was performed on the Illumina NovaSeq X Plus platform (Illumina Inc., San Diego, CA, USA) with a paired-end 150 bp (PE150) strategy.

Raw reads were filtered using fastp (v0.23.2) [27] to remove adapters and low-quality bases, yielding high-quality clean reads. Because no chromosome-level reference genome is available for *P. corylifolia*, a de novo transcriptome assembly strategy was adopted. Clean reads from all samples were pooled and assembled using Trinity (v2.15.1) [28] with default parameters. To reduce redundancy, Corset (v1.09) [29] software was employed for hierarchical clustering of transcripts based on shared read alignments and expression patterns. Specifically, Corset assigns transcripts sharing the same reads to a super-cluster (designated by an integer ID), within which further clustering generates sub-clusters (designated by a decimal number). For each sub-cluster, the longest transcript was retained as a Unigene, named in the format “Cluster_XXXXX.Y,” where “XXXXX” represents the Corset super-cluster ID and “Y” the sub-cluster number. A total of 80,402 Unigenes were obtained, with an N50 of 2998 nt and a mean length of 2251 nt. The total assembled bases were 180,959,590 nt. Assembly completeness was evaluated using BUSCO (https://busco.ezlab.org, accessed on 10 October 2025) [30] with the embryophyta_odb10 database (Figure S2).

For expression quantification, clean reads from each sample were aligned back to the assembled Unigene reference using RSEM (v1.3.1) [31]. Expression levels were calculated as FPKM for visualization and WGCNA purposes. For differential expression analysis, raw RSEM expected counts were supplied to DESeq2 (v1.22.2) [32]. Since all sample groups contained three biological replicates, the default DESeq2 workflow was applied. The Benjamini–Hochberg method was applied to control the false discovery rate (FDR). Genes with |log_2_ fold change| ≥ 1 and FDR < 0.05 were defined as differentially expressed genes (DEGs).

For functional annotation, Unigene sequences were searched against the KEGG, NR, Swiss-Prot, GO, COG/KOG, and TrEMBL databases using DIAMOND (v2.0.9) [33] BLASTX (E-value threshold: 1 × 10^−5^). Conserved protein domains were identified by HMMER [34] searches against the Pfam database. Genes were assigned to metabolic pathways based on KEGG pathway annotations. Transcription factors were identified and classified using iTAK (v1.7a) [35] software, which utilizes the classification rules from the PlnTFDB and PlantTFDB databases via hmmscan alignment.

### 2.5. Joint Transcriptomic and Metabolomic Investigation

To examine tissue-specific associations in secondary-metabolite biosynthesis in *P. corylifolia*, an integrated analysis of transcriptomic and metabolomic data was performed. Differentially accumulated metabolites (DAMs) and differentially expressed genes (DEGs) were identified according to the criteria described in Section 2.3.3 and Section 2.4, respectively.

#### 2.5.1. Tissue-Specific Expression Profiling of Target Pathway Genes and Metabolites

Four KEGG pathways related to coumarin and flavonoid biosynthesis were selected, including ko00940 (phenylpropanoid biosynthesis), ko00941 (flavonoid biosynthesis), ko00943 (isoflavonoid biosynthesis), and ko00944 (flavone and flavonol biosynthesis). Based on the KEGG pathway annotations, key genes and metabolites within these pathways were identified from the lists of differentially expressed genes and differentially accumulated metabolites. Pathway schematic diagrams were drawn using Adobe Illustrator (version 2026). Heatmaps showing the expression abundance of pathway genes (FPKM) and metabolites (relative peak area) across different tissues were generated using the WeChat online bioinformatics platform (https://www.bioinformatics.com.cn, accessed on 4 February 2026).

#### 2.5.2. Weighted Gene Co-Expression Network Analysis (WGCNA)

Gene expression values (FPKM) from all five tissues were used for WGCNA [36], starting from the full reference set of 80,402 Unigenes. Gene filtering and network construction were performed using TBtools (WGCNAshiny module) (version 2.441) [37]. First, low-confidence genes were removed by filtering out those with counts less than 1 in more than 90% of the samples, leaving 20,270 genes. The top 15,000 genes with the highest median absolute deviation (MAD) were then selected as the input gene set for network construction, resulting in a final set of 14,998 genes for network construction. Based on the scale independence plot and the information in the sft table (Table S5), a soft-thresholding power of 26 was selected (scale-free topology fit R^2^ = 0.79) (Figure S3). The topological overlap matrix (TOM) was calculated using the signed network type, and modules were identified by dynamic tree cutting with a minimum module size of 30. Similar modules were merged at a cut height of 0.25 (mergeCutHeight = 0.25). A module hierarchical clustering dendrogram was generated in TBtools. The module–metabolite–trait correlation heatmap was created using R (pheatmap package) (version 4.5.2). Modules significantly correlated with target tissues and metabolites were selected for downstream analyses. For module–trait correlations and TF–pathway gene correlations, the Benjamini–Hochberg (BH) procedure was applied to control the false discovery rate (FDR). Both nominal and FDR-adjusted *p*-values are reported in Supplementary Table S6.

#### 2.5.3. Construction of TF–Pathway Gene Co-Expression Network and Screening of Hub Transcription Factors

To identify transcription factors within the target module that were associated with flavonoid and coumarin biosynthesis, TFs were first screened from the module genes based on iTAK annotations (Section 2.4). Pearson correlation coefficients were then calculated between each TF and all structural genes in the flavonoid and coumarin pathways using FPKM values from all tissue samples. Significant co-expression pairs were retained at |r| ≥ 0.8 and *p* < 0.05, and a co-expression network was constructed using Cytoscape (version 3.9.1) [38]. The degree of a node was defined as the number of directly connected edges. Additionally, intramodular connectivity (Kwithin) and whole-network connectivity (Ktotal) were calculated for each TF based on WGCNA results. Candidate hub TFs were prioritized by integrating degree, Kwithin, and Ktotal.

All statistical analyses were performed in R, and figures were finalized using Adobe Illustrator.

## 3. Results

### 3.1. Metabolite Profiling of P. corylifolia

**Figure 1:**
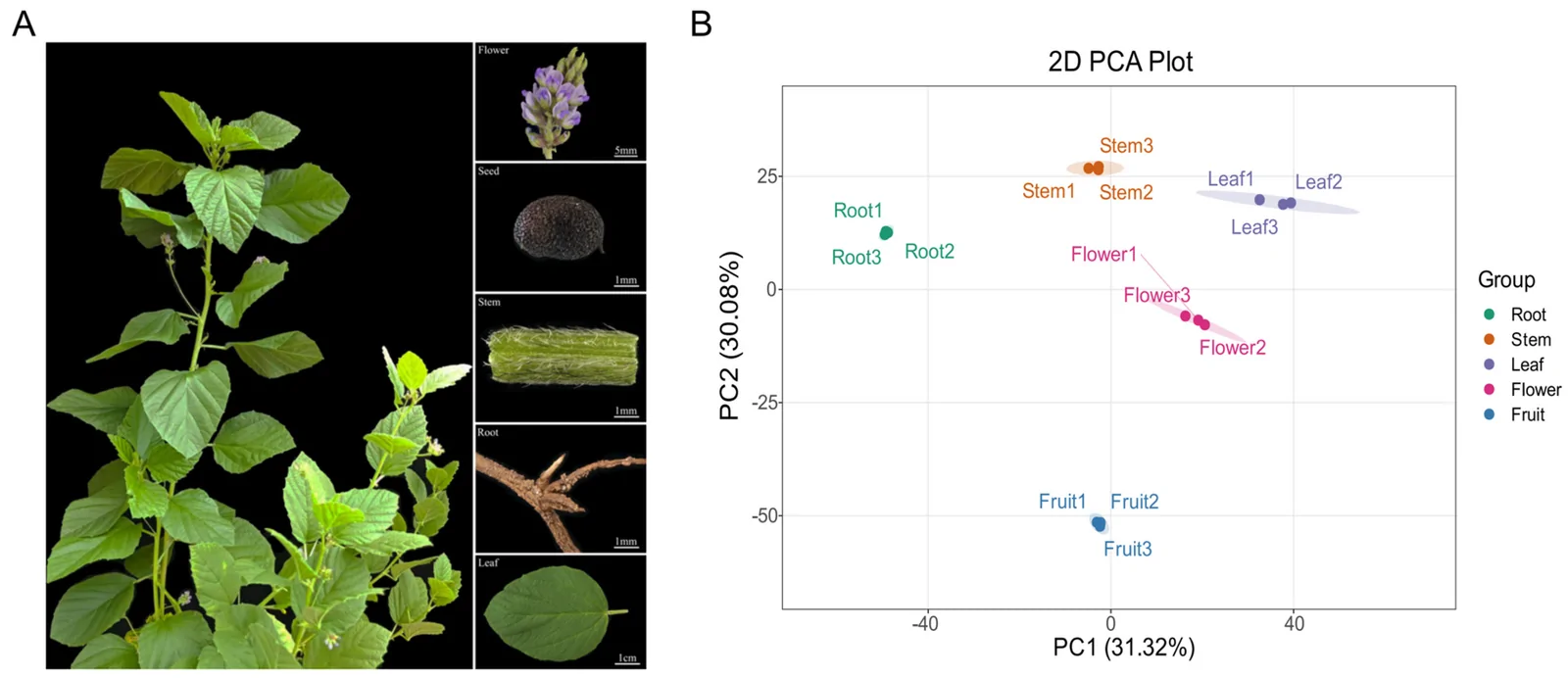
Overview of the phenotypic and metabolomic data for *P. corylifolia*. (**A**) Phenotypic profiles of five parts of *P. corylifolia*. (**B**) Principal component analysis (PCA) of metabolite distribution.

To characterize the tissue-specific distribution patterns of secondary metabolites in *P. corylifolia*, we employed widely targeted metabolomics to analyze five tissue types: roots, stems, leaves, flowers, and fruits (Figure 1A).

Metabolomic data underwent rigorous quality control (QC) assessment, with Pearson correlation coefficients > 0.99 among QC samples and >85% of metabolites having a CV < 0.5, indicating good data stability and reproducibility (Figure S4). To evaluate the reproducibility of the metabolomic data, principal component analysis (PCA) was conducted for unsupervised clustering of the global metabolite profiles. The five tissue types of *P. corylifolia* showed distinct intergroup separation along the PC1 (31.32%) and PC2 (30.08%) axes (Figure 1B). A heatmap of inter-sample correlations further demonstrated exceptionally strong Pearson correlation estimates (r > 0.98) among biological replicates within the same tissue, whereas markedly lower correlations were observed between distinct tissues (Figure S5). These findings indicated high within-tissue reproducibility and pronounced tissue-specific metabolic profiles. Notably, the fruit, which is the primary medicinal part, differed markedly from the other tissues (root, stem, leaf, and flower). Ultra-high-performance liquid chromatography-tandem mass spectrometry (UPLC-MS/MS) was employed for targeted metabolomic profiling, a well-established analytical technique extensively applied in plant metabolomics research [39]. Using this approach, we analyzed the metabolite composition of *P. corylifolia*, and successfully identified a total of 2830 distinct compounds (Table S3). Among these, flavonoids (653 compounds) constituted the largest class of annotated metabolites, followed by terpenoids (376 compounds), lignans (118 compounds) and coumarins (76 compounds). The overall proportion of each chemical class is shown in Figure S6, and the numbers of metabolites in each chemical class are listed in Table S7.

### 3.2. Analysis of Key Metabolites in P. corylifolia

**Figure 2:**
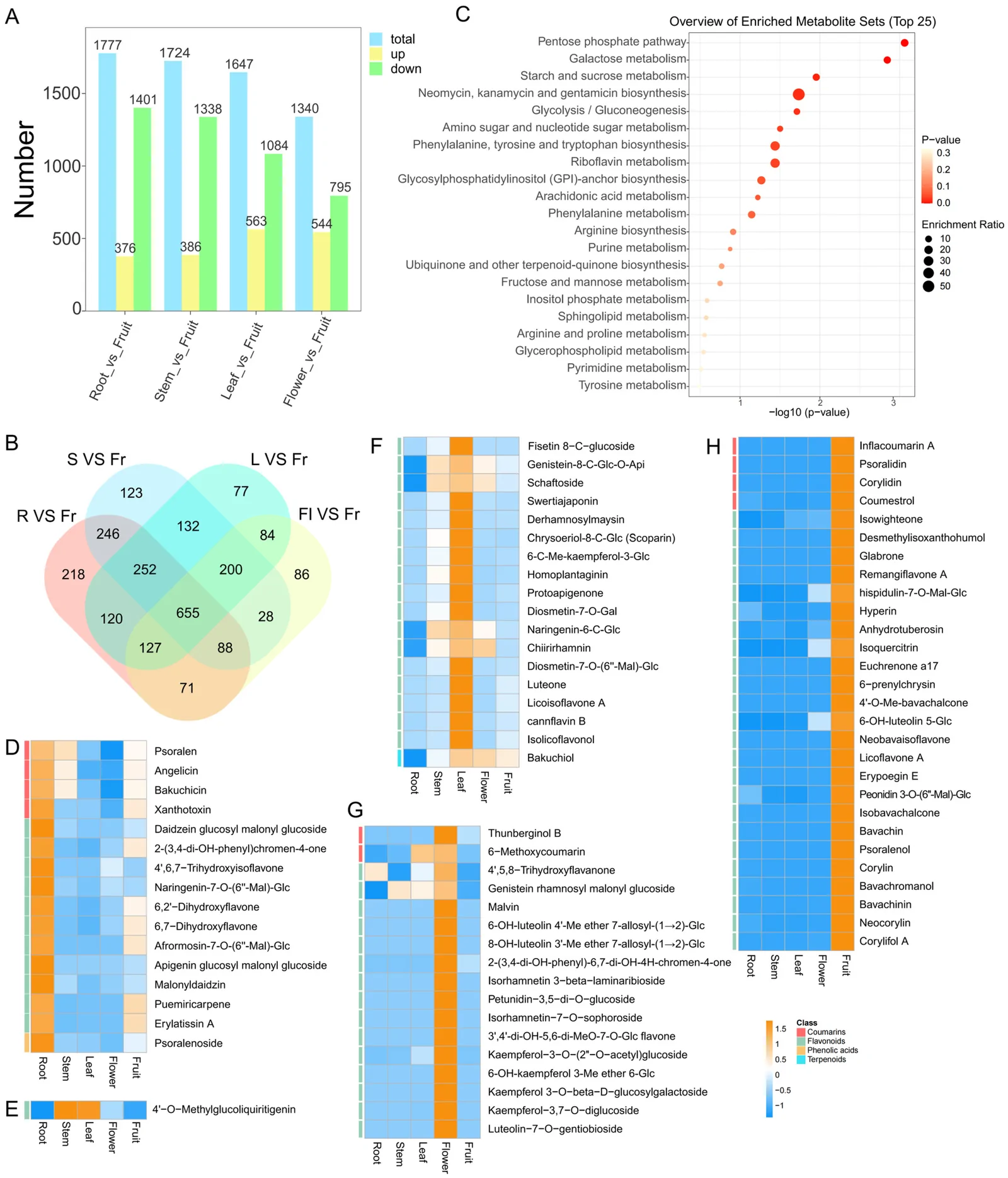
Analysis of differential metabolites in various parts of *P. corylifolia*. (**A**). Statistics on the abundance of upregulated and fruit-enriched DAMs in each part compared to the fruit. (**B**) Venn diagram of differential metabolites. (**C**) KEGG pathway enrichment analysis of fruit-enriched DAMs. (**D**–**H**) Accumulation heatmaps of the 81 metabolites in five tissues (root, stem, leaf, flower and fruit).

Because the fruit is the medicinal part of *P. corylifolia*, we focused on differential metabolites in other parts compared to the fruit. In the four comparison groups—“root vs. fruit,” “stem vs. fruit,” “leaf vs. fruit,” and “flower vs. fruit”—fruit-enriched differentially accumulated metabolites (fruit-enriched DAMs; i.e., metabolites with lower abundance in other tissues but higher abundance in fruit, negative log_2_FC in “other tissue vs. fruit” comparisons) outnumbered upregulated ones in each group, with the root vs. fruit group exhibiting the highest total number of differential metabolites (Figure 2A). Collectively, 655 common differentially accumulated metabolites were identified (Figure 2B, Table S8). To identify the core metabolites that were enriched in *P. corylifolia* fruits, we examined the fruit-enriched DAMs across the four comparison groups, revealing 423 shared fruit-enriched DAMs (Figure S7). Among the 423 fruit-enriched DAMs, 98 metabolites with KEGG annotations were subjected to KEGG pathway enrichment analysis (Figure 2C, Table S9). These metabolites were enriched in several pathways, including and phenylalanine, tyrosine, and tryptophan biosynthesis, which are upstream of the flavonoid and coumarin biosynthesis pathways. Consistently, the metabolite enrichment analysis of the same 98 annotated metabolites (Figure S8, Table S10) revealed significant enrichment of compound classes directly related to the bioactive constituents of *P. corylifolia*, particularly coumarins and flavonoids. This result is consistent with the KEGG enrichment analysis shown in Figure 2C.

To comprehensively display tissue-specific accumulation patterns across all five tissues, we selected 80 unique metabolites that included a set of well-characterized bioactive components of *P. corylifolia* [4,40,41] and other metabolites with high total ion intensity, and generated metabolite accumulation heatmaps based on their relative contents in each tissue (Figure 2D–H, Table S11). Hierarchical clustering of these heatmaps revealed distinct tissue-preferential metabolite clusters: Psoralenoside and Bakuchicin were enriched in roots; Swertiajaponin and Luteone were enriched in leaves; Isobavachalcone, Bavachin, and Corylin were enriched in fruits.

### 3.3. Transcriptomic Characterization of P. corylifolia

**Figure 3:**
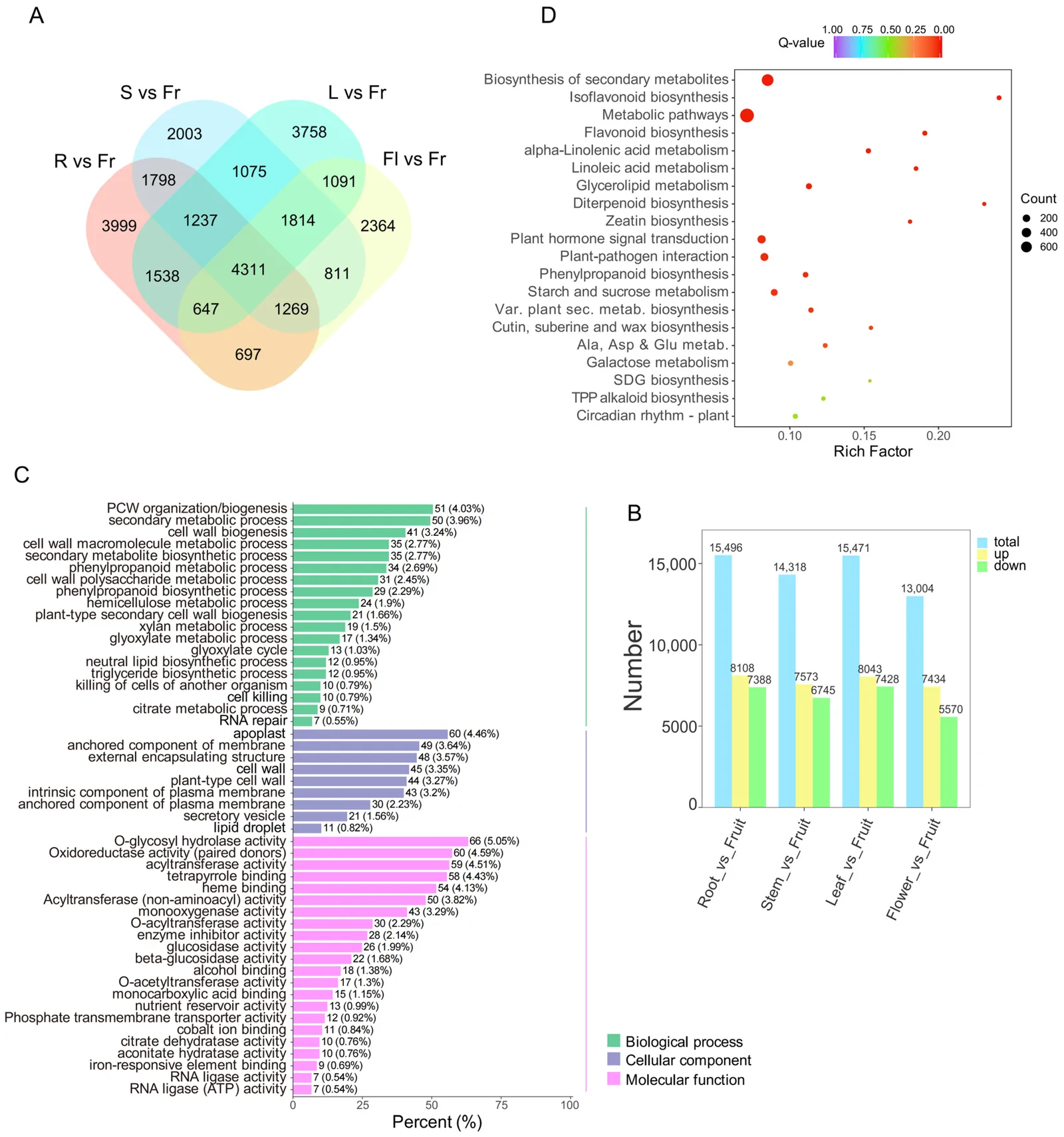
Analysis of differentially expressed genes in different parts of *P. corylifolia*. (**A**) Venn diagram of differentially expressed genes. (**B**) Statistics on the number of up- and down-regulated genes. (**C**) GO functional annotation enrichment of dysregulated transcripts. (**D**) KEGG pathway enrichment analysis of differentially expressed genes. Rich factor represents the ratio of the number of differentially expressed genes annotated to a given pathway to the total number of genes annotated to that pathway.

Transcriptomic sequencing yielded a total of 127.51 Gb of clean data, with a Q30 base percentage exceeding 97% and 80,402 unigene sequences (Table S12). The overall mapping rates of the 15 samples ranged from 94.11% to 94.90% (Table S13). A total of 4681 putative TFs were predicted from the full set of 80,402 Unigenes without additional filtering for fragment length or sequence completeness. Among all Unigenes, 84.88% were annotated in at least one database, with annotation rates as follows: NR 84.27%, GO 74.34%, KEGG 66.23%, Swiss-Prot 64.00%, Pfam 63.89%, TrEMBL 83.88%, and KOG 51.44% (Table S14). To assess the transcriptomic data consistency, principal component analysis (PCA) and sample correlation analysis were conducted on the transcriptomic data from the five tissues of *P. corylifolia* (Figure S9). These analyses identified that biological replicates from the same part clustered closely together, while samples from different parts were clearly separated. Collectively, PC1 and PC2 accounted for 44.7% of the total variation, revealing that each tissue exhibited a distinct gene-expression pattern and good intra-group reproducibility, providing a reliable data foundation for subsequent differential gene expression and co-expression network analyses. Transcriptome comparison analyses identified 15,546, 14,318, 15,471, and 13,004 differentially expressed genes respectively in the root, stem, leaf and flower samples compared to the fruit samples (Figure 3B). In all pairwise comparisons, the number of upregulated genes exceeded the number of downregulated genes, suggesting that more genes exhibited increased expression levels in these tissues (Figure 3B). Across the four tissue comparisons, a total of 4311 common differentially expressed genes were identified (Figure 3A and Table S15).

Gene Ontology (GO) enrichment analysis was then performed on these 4311 common DEGs (Figure 3C, Table S16). At the biological process level, these genes were enriched in terms including the phenylpropanoid metabolism pathway, phenylpropanoid biosynthesis pathway, secondary metabolite biosynthesis pathway, and hemicellulose metabolism pathway. Among these, the phenylpropanoid pathway is the core upstream pathway for the biosynthesis of coumarins and flavonoids; its enrichment suggests an association between these transcriptional changes and active-component biosynthesis in *P. corylifolia*. KEGG pathway enrichment analysis of the same 4311 common DEGs (Figure 3D) revealed that they were markedly enriched in a set of key pathways, including secondary metabolite biosynthesis, isoflavonoid biosynthesis, flavonoid biosynthesis, and phenylpropanoid biosynthesis.

We analyzed transcriptional changes across the four tissue groups and annotated the TFs in the transcriptomic dataset. A total of 4681 TFs (divided into 89 families) were annotated (Table S17). A total of 1419, 1242, 1175, and 1105 differentially expressed transcription factors (DETFs) were detected in root vs. fruit, stem vs. fruit, leaf vs. fruit, and flower vs. fruit, respectively (Table S18). Among these, the AP2/ERF-ERF family was the most abundant, followed by the bHLH, MYB-related, and bZIP families (Figure S10).

### 3.4. Integrated Transcriptomic and Metabolomic Analysis

**Figure 4:**
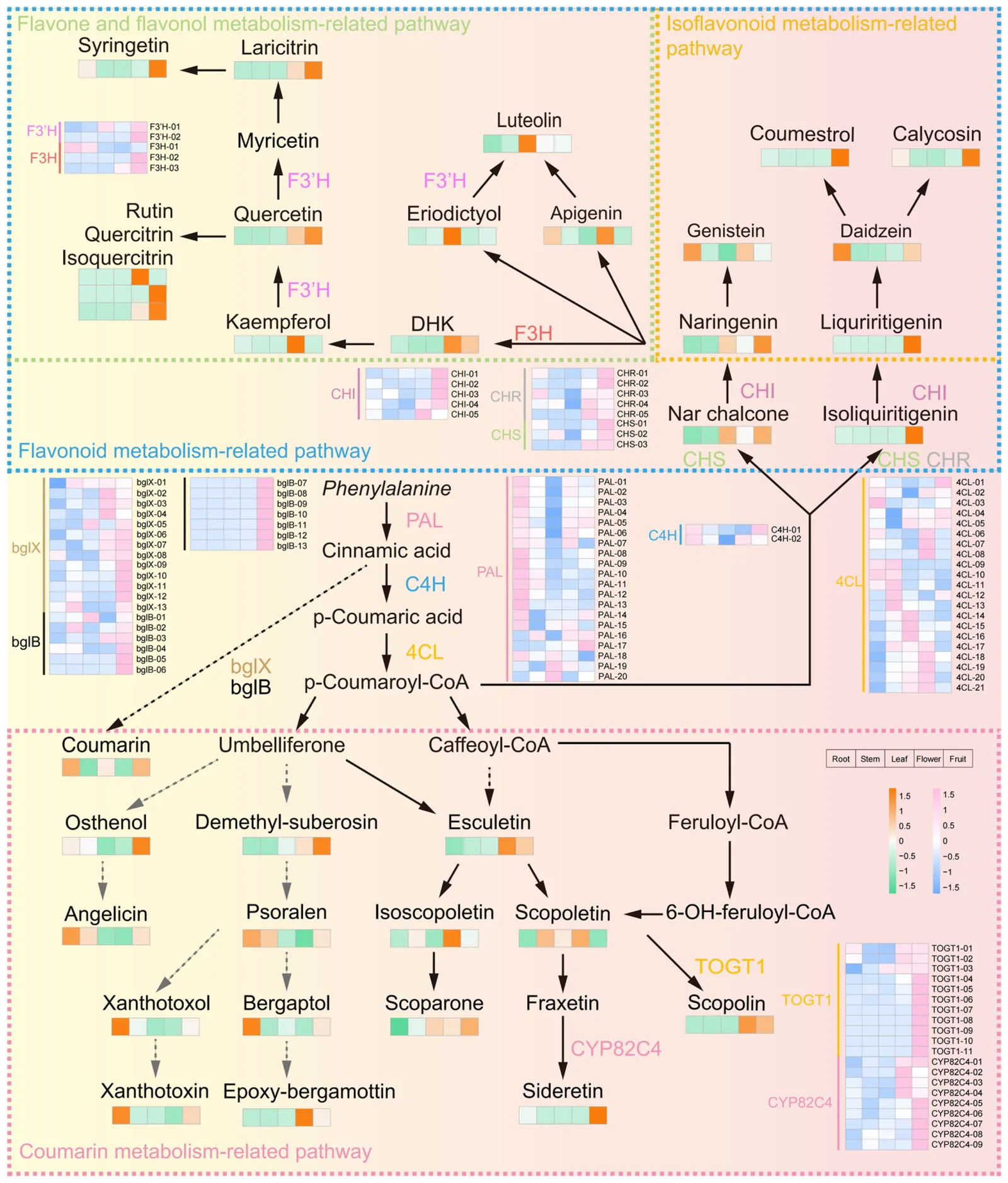
Metabolite abundance and putative gene expression in flavonoid and coumarin pathways. The figure illustrates the core reaction backbone of phenylpropanoid-derived flavonoid and coumarin metabolism. The pathway backbone represents well-established biochemical reactions conserved in plants. The assignment of unigenes to each catalytic step is putative, based on KEGG annotation and homologous sequence alignment. Black dashed arrows denote multi-step reactions for which individual enzymatic steps are not shown. Grey dashed arrows denote the downstream modification steps of the furanocoumarin branch, which are hypothetical and inferred from studies in other species. The orange and green heatmaps represent relative metabolite levels, while the pink and blue heatmaps represent the relative expression abundance of putative enzyme genes; orange indicates high metabolite abundance, whereas pink indicates high gene expression. The samples are, in order: root, stem, leaf, flower, and fruit. The original FPKM values of putative enzyme genes, the relative metabolite abundances, and the detailed annotation information are provided in Supplementary Tables S21, S22, and S20, respectively.

To characterize tissue-specific expression patterns associated with the biosynthesis of characteristic secondary metabolites in *P. corylifolia*, we mapped unigenes to KEGG pathways related to coumarin and flavonoid biosynthesis, including ko00940 (phenylpropanoid biosynthesis), ko00941 (flavonoid biosynthesis), ko00943 (isoflavonoid biosynthesis), and ko00944 (flavone and flavonol biosynthesis) pathways, as well as their metabolites, to analyze tissue-specific expression patterns (Figure 4).

The core backbone of these pathways is well established in plants: it begins with phenylalanine, and through the catalysis of conserved key enzymes such as PAL, C4H, and 4CL, generates p-coumaroyl-CoA, which subsequently branches into the flavonoid and coumarin metabolic pathways. The assignment of unigenes to these catalytic steps is putative and is based on sequence homology to functionally characterized enzymes. The PAL gene family was predominantly expressed in roots; the 4CL family showed higher expression in stems, leaves and flowers; only two C4H isoforms were annotated, with no obvious tissue-specific expression difference.

In the flavonoid metabolism branch, some putative core enzyme genes, particularly certain CHS and CHI members, showed relatively high expression in fruits, which was broadly consistent with the accumulation of several flavonoid metabolites in fruit tissues. However, the correlation strength varied among gene family members, and not all individual members exhibited significant tissue-specific associations with a single metabolite (Table S19). For the flavone and flavonol sub-branch, the expression patterns of putative F3’H genes varied among members; for example, F3H01 was predominantly expressed in roots and stems, while its expression in fruits was relatively low. At the metabolite level, quercetin was enriched in fruits, whereas kaempferol and apigenin were enriched in flowers, and luteolin was enriched in leaves. The negative correlation between F3H01 and quercetin (r = −0.666, Table S19) suggests that this particular F3’H member may not be directly responsible for the fruit-specific accumulation of quercetin, or that additional regulatory mechanisms are involved. For the isoflavonoid branch, few key enzyme genes were annotated in this study, and no corresponding gene expression heatmap is displayed in Figure 4. At the metabolite level, daidzein and genistein were mainly enriched in roots, while coumestrol was enriched in fruits.

In the coumarin metabolic pathway, the simple coumarin branch and furanocoumarin branch showed distinct tissue distribution characteristics. Putative TOGT1 genes were mainly highly expressed in fruits, corresponding to the high accumulation of scopolin in flowers and fruits. For the furanocoumarin sub-branch, putative CYP82C4 genes were highly expressed in flowers and fruits. At the metabolite level, psoralen and xanthotoxin were mainly enriched in roots.

Overall, the tissue-specific gene expression patterns showed clear correlative trends with the tissue-specific accumulation of flavonoids and coumarins, providing candidate gene targets for further functional investigation of biosynthetic and regulatory mechanisms. Detailed functional annotation information for all pathway genes is provided in Supplementary Table S20; the original FPKM values of genes and the relative abundance data of metabolites in Figure 4 are listed in Supplementary Table S21 and Table S22, respectively.

### 3.5. WGCNA Co-Expression Network Analysis and Screening of Hub Transcription Factors

**Figure 5:**
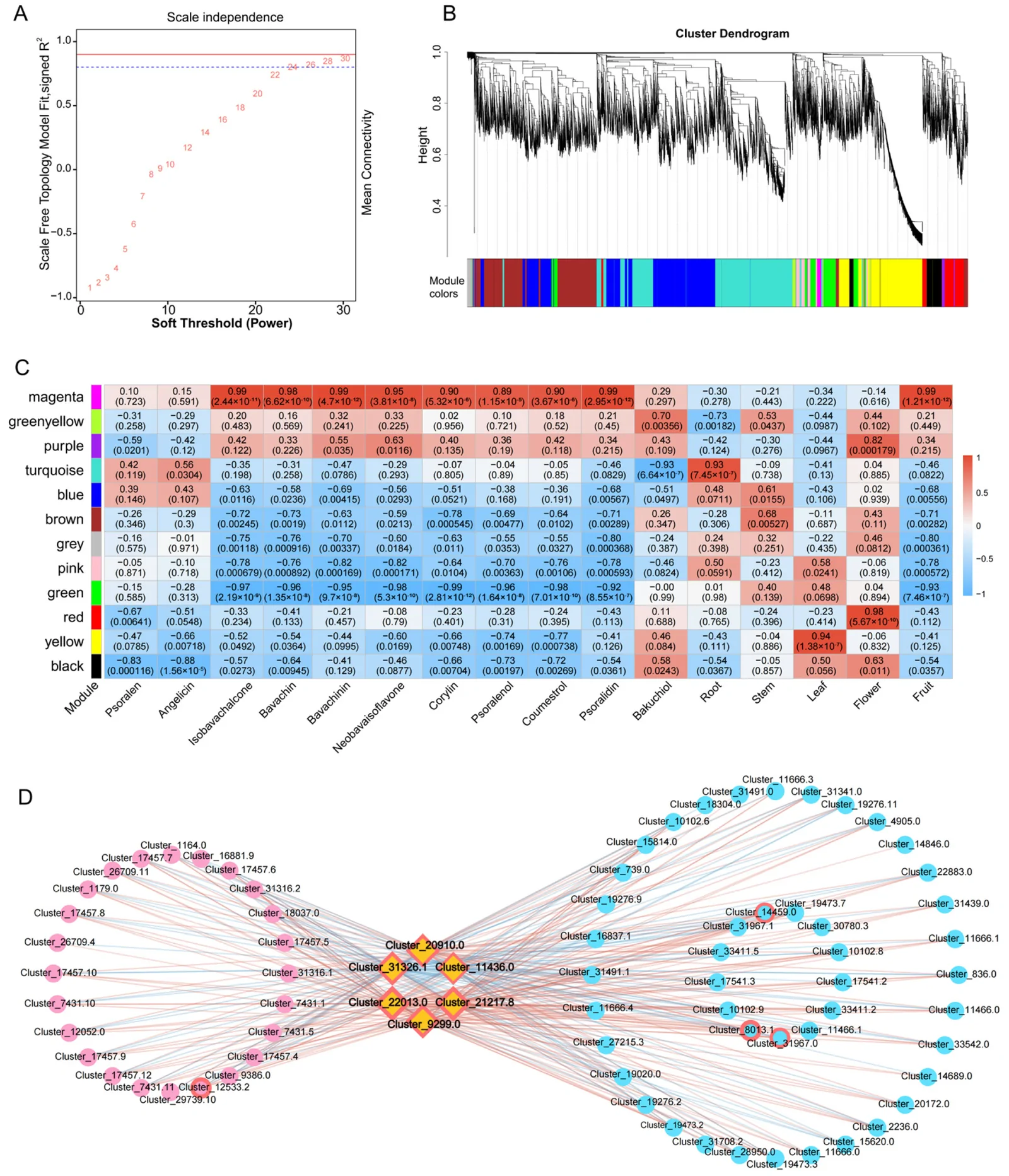
WGCNA of transcriptomic traits and flavonoid and coumarin metabolites in *P. corylifolia*. (**A**) Soft-threshold selection analysis to determine the optimal soft threshold for constructing a scale-free network (blue dashed line: R^2^ = 0.8; red line: R^2^ = 0.9); (**B**) Hierarchical clustering dendrogram of genes and module assignment. The y-axis indicates clustering height, a dimensionless value reflecting the topological overlap dissimilarity between gene clusters. Distinct colors below the dendrogram correspond to different co-expression modules.; (**C**) Module–metabolite–tissue correlation heatmap. Each cell shows two values: the upper value is the Pearson correlation coefficient between the module eigengene and the corresponding tissue or metabolite, and the lower value (in parentheses) is the FDR-adjusted *p*-value; (**D**) Co-expression network of TFs from the magenta module with flavonoid and coumarin pathway genes (344 edges, |r| ≥ 0.8, *p* < 0.05). Node colors: orange, TFs; blue, flavonoid pathway genes; pink, coumarin pathway genes. Edge colors: red, significant positive correlation; blue, significant negative correlation. The size of the red outline around each TF node is mapped to intramodular connectivity (Kwithin). Node degree is defined as the number of directly connected edges.

Based on integrated transcriptomic and metabolomic datasets, weighted gene co-expression network analysis (WGCNA) was performed using expression data from all 15 samples (five tissues × three biological replicates) combined across tissues. Soft-threshold screening was performed by jointly evaluating scale-free topology fit and mean network connectivity. When power = 26, the scale-free topology fit R^2^ reached 0.79 with a mean connectivity of 199.03, representing the optimal balance between network connectivity and scale-free topology under this dataset. The co-expression network was therefore constructed using this parameter (Figure 5A, Table S5). All genes were categorized into 12 co-expression modules (Figure 5B). Gene counts per module ranged from 95 to 3580.

The module–metabolite–tissue correlation heatmap showed that the green-yellow and turquoise modules were significantly positively correlated with roots, the blue module with stems, the brown module with leaves, the black module with flowers, and the magenta module with fruits (FDR-adjusted *p* < 0.05). Additionally, the magenta module exhibited positive correlations with various coumarin and flavonoid metabolites, including corylin, psoralidin, and coumestrol, indicating that it is a candidate co-expression module associated with these metabolites in fruits (Figure 5C).

To further examine candidate regulatory associations, TFs were screened from the magenta module (129 genes), and Pearson correlation coefficients were calculated between each TF and all structural genes in the flavonoid and coumarin pathways (Table S23). A TF–pathway gene co-expression network was constructed using thresholds of |r| ≥ 0.8 and FDR-adjusted *p* < 0.05 (Figure 5D). The network comprised 344 significant edges involving six TFs. Based on node degree, intramodular connectivity (Kwithin), and whole-network connectivity (Ktotal), the six TFs were classified into three candidate-priority tiers: (1) hubs, including Cluster_22013.0 (C3H; best homolog: rice OsC3H30) and Cluster_21217.8 (NF-YA; *Arabidopsis* homolog NFYA9/AT3G20910); (2) positively correlated candidates, including Cluster_9299.0 (Rcd1-like) and Cluster_20910.0 (NAC; *Arabidopsis* homolog NAC002/AT5G04410); (3) negatively correlated candidates, including Cluster_31326.1 (bZIP; homolog CPRF2) and Cluster_11436.0 (C2H2; *Arabidopsis* homolog JJJ1/AT1G74250), both of which exhibited exclusively negative correlations (Table S24).

## 4. Discussion

*Psoralea corylifolia* is a well-documented medicinal legume whose dried ripe fruits serve as the official medicinal part in traditional clinical practice. While the pharmacological activities of its characteristic flavonoids and furanocoumarins have been extensively investigated [4,5,6,7,8], the molecular basis underlying the tissue-specific accumulation of these bioactive compounds remains largely unexplored. In this study, we integrated widely targeted metabolomics and transcriptomics across five tissues (root, stem, leaf, flower, and fruit) of *P. corylifolia*, combined with weighted gene co-expression network analysis, to systematically characterize tissue-specific metabolic and transcriptional profiles. This work is primarily a candidate-screening study rather than a functional verification of regulatory mechanisms: we identified co-expression modules and candidate transcription factors associated with the high accumulation of flavonoids and coumarins in fruits. These findings provide a valuable data resource and candidate gene pool for subsequent functional validation of biosynthetic pathways and regulatory networks in *P. corylifolia*.

### 4.1. Tissue-Specific Accumulation of Secondary Metabolites Across Organs

Plant secondary metabolism is tightly coupled with organ functional differentiation, with distinct tissues adopting specialized metabolic profiles to support their physiological roles [42]. In this study, unsupervised clustering of both metabolomic and transcriptomic data showed clear separation among the five tissue types, with fruits exhibiting the most distinct profile compared with vegetative tissues (Figure 1B and Figure S7). This pattern of organ-specific metabolic remodeling is widely observed in medicinal plants. For example, the major bioactive flavonoid baicalin in *Scutellaria baicalensis* is predominantly accumulated in roots, consistent with its root-based medicinal use [43,44]; flavonoid glycosides in *Carthamus tinctorius* are specifically enriched in floral tissues, corresponding to its floret medicinal part [45,46]; and terpenoid bioactive compounds in *Amomum tsao-ko* are highly concentrated in fruits [47]. In this study, the preferential accumulation of isobavachalcone, bavachin and corylin in *P. corylifolia* fruits is consistent with reports that bioactive constituents can be enriched in the officinal organs of medicinal plants.

For leguminous species, organ-specific isoflavonoid accumulation has been associated with tissue-specific MYB transcription factors. For instance, in soybean (*Glycine max*), isoflavones predominantly accumulate in seeds and function as defensive secondary metabolites and nutritional components [48]; their seed-specific accumulation pattern is transcriptionally controlled by seed-specific MYB transcription factors [22]; *Medicago truncatula* exhibits root-specific flavonoid biosynthesis controlled by MYB40 [21]. In this study, the fruit-specific enrichment of prenylated flavonoids in *P. corylifolia* extends this paradigm, reflecting both conserved flavonoid tissue distribution patterns and species-specific structural modification characteristics in legumes.

With respect to coumarin distribution, our data detected higher psoralenoside and psoralen levels in roots, which is partially inconsistent with a previous quantification study reporting maximum psoralenoside abundance in fruits, as that study only examined stems, leaves, flowers, and fruits [21]. Such discrepancies may stem from multiple factors, including differences in germplasm sources, cultivation environments, developmental stages at sampling, and soil microbial communities [49]. The earlier study only quantified limited target compounds via UHPLC-PDA, whereas our widely targeted metabolomics covers a broader range of metabolites. Furthermore, tissue-specific transformation and translocation of coumarin glycosides during plant development may also lead to inconsistent distribution results across independent investigations [50].

### 4.2. Concordant Enrichment of the Phenylpropanoid Pathway at Transcript and Metabolite Levels

The phenylpropanoid pathway serves as the shared upstream route for both flavonoid and coumarin biosynthesis [13,14]. In the present study, differentially accumulated metabolites and differentially expressed genes were both enriched in phenylpropanoid biosynthesis, flavonoid biosynthesis and isoflavonoid biosynthesis pathways (Figure 2C and Figure 3D). Such pathway-level consistency between transcriptional variation and metabolite abundance has also been documented in multi-tissue multi-omics studies of other medicinal plants. For example, root-specific expression of phenylpropanoid and flavonoid structural genes in *S. baicalensis* coincides with root-specific accumulation of baicalin and wogonoside [43,45]; upregulated flavonoid biosynthetic genes in safflower florets match the high accumulation of flavonoid glycosides in flowers [45,46].

It should be emphasized that this pathway-level concordance reflects correlative trends and cannot demonstrate regulatory interactions between individual genes and metabolites. In our data, in pairwise comparisons between vegetative tissues and fruits, the number of upregulated genes was higher than that of upregulated metabolites (Figure 2A and Figure 3B). This phenomenon may be associated with multi-layered regulation including transcriptional control, post-translational modification of biosynthetic enzymes, and inter-tissue translocation of metabolic intermediates [51,52]. Nevertheless, without isotope tracing, spatial metabolomics or transporter functional evidence, we avoid overinterpreting this pattern to deduce source-sink transport relationships.

### 4.3. Candidate Co-Expression Modules and Transcription Factors Associated with Fruit-Specific Metabolism

Weighted gene co-expression network analysis is a common bioinformatics tool to screen gene modules linked to phenotypic traits. Importantly, this method identifies correlation rather than causal regulatory relationships [36]. In our WGCNA results, 12 co-expression modules were obtained, among which the magenta module exhibited significant positive correlations with fruit tissue and multiple flavonoid and coumarin metabolites (Figure 5C), representing a key candidate module related to fruit secondary metabolism.

We further screened transcription factors within the magenta module and constructed a co-expression network between transcription factors and flavonoid/coumarin pathway structural genes. Six candidate TFs were identified and classified into three candidate-priority tiers based on network topology metrics (Figure 5D, Table S24). Unlike many legume studies highlighting MYB as master regulators of flavonoid biosynthesis [21,22], our hub TFs belong to C3H and NF-YA families. Previous studies have confirmed that NF-Y family participates in plant flavonoid regulation by binding to CCAAT elements in the promoters of key biosynthetic genes such as CHS [53,54]; the identified NF-YA candidate broadens the set of TFs for future investigation in legume fruit secondary metabolism. Two candidate TFs (bZIP and C2H2) displayed exclusively negative correlations with pathway genes, a pattern consistent with previously reported negative regulators in other species. Negative regulators of isoflavonoid biosynthesis have also been reported in soybean, where C2H2-type zinc-finger TFs can modulate metabolite accumulation by repressing pathway gene expression [55]. Compared with transcriptional activators, repressors of medicinal plant active component biosynthesis are less characterized, yet they may provide targets for future functional studies.

We stress that all candidate transcription factors were screened for their potential association with flavonoid and coumarin accumulation, solely on the basis of co-expression correlation. Whether these TFs can directly bind to promoters of biosynthetic genes and activate/repress transcription requires further experimental verification. Without yeast one-hybrid, dual-luciferase assay, transient or stable genetic transformation evidence, these candidates should not be regarded as confirmed regulatory factors.

### 4.4. Research Limitations and Future Perspectives

Several limitations of this study should be acknowledged. First, a chromosome-level reference genome for *P. corylifolia* is unavailable; we adopted de novo transcriptome assembly, which may lead to incomplete gene annotation, loss of isoform information and paralog misclassification [56]. High-quality reference genome data will greatly improve pathway dissection and regulatory network analysis in future work.

Second, this study employed a blocked paired sampling design, with all tissue replicates derived from the same set of plant replicate pools. The current univariate differential analysis did not incorporate the replicate pool as a blocking covariate. However, unsupervised PCA and sample correlation analyses both demonstrate that inter-tissue variation far exceeds inter-pool variation within the same tissue, and core findings—including pathway enrichment results and WGCNA module assignments—are highly stable, indicating that the main biological conclusions are not substantively affected.

Third, all conclusions on candidate genes and modules originate from bioinformatic correlation analysis; no experimental validation including RT-qPCR verification, protein–DNA interaction detection or genetic functional tests was performed in this work.

Fourth, the widely targeted metabolomic analysis carries several inherent methodological limitations. Metabolite annotations were obtained through spectral matching against a commercial database containing pre-validated standard spectra, and no authentic reference standards were analyzed concurrently with the experimental samples. Quantification was based on relative MRM peak areas, which are valid for comparing the same metabolite across different samples but cannot be summed across chemically distinct compounds or interpreted as absolute concentrations.

Fifth, the WGCNA is constrained by the limited sample size. Only 15 transcriptome samples from five tissues (three replicates per tissue) were included in network construction, and the scale-free topology fit R^2^ at the selected soft threshold was slightly below the conventional 0.80 reference value [36]. Although sensitivity analysis confirmed the stability of core results, the statistical power of the co-expression network remains limited, and the candidate modules and prioritized TFs require further validation in larger sample cohorts and independent experimental systems. Future studies with larger sample sizes should include more rigorous resampling-based stability analyses (e.g., bootstrap or leave-one-replicate-out) to further validate the module assignments and TF prioritization.

Future research directions include: verifying expression patterns of key structural genes and candidate TFs via RT-qPCR; characterizing physical interactions and regulatory effects between TFs and pathway promoters using yeast one-hybrid and dual-luciferase reporter assays; and preliminarily testing gene functions using the Nicotiana benthamiana transient expression system [57]. Ultimately, establishing a stable genetic transformation system of *P. corylifolia* will be essential to fully clarify the biosynthetic and regulatory network of its bioactive compounds.

In summary, this study establishes a multi-tissue metabolome and transcriptome dataset of *P. corylifolia*. Via integrated bioinformatic analysis, we screened candidate co-expression modules and transcription factors related to fruit-specific flavonoid and coumarin accumulation. Although the regulatory functions of these candidates await experimental validation, our results provide fundamental data resources and a candidate framework for follow-up functional genomics research on this important medicinal plant.

## 5. Conclusions

In this study, we constructed a multi-tissue metabolome and transcriptome dataset for *P. corylifolia* and systematically characterized the tissue-specific accumulation patterns of flavonoids and coumarins. By integrating weighted gene co-expression network analysis with a TF–pathway gene co-expression strategy, we identified the magenta module as a candidate module associated with the fruit-specific enrichment of bioactive compounds. From this module, six candidate transcription factors were screened and classified into three candidate-priority tiers—hubs, positively correlated TFs, and negatively correlated TFs—based on network topological metrics and intramodular connectivity. Among these, Cluster_22013.0 (C3H) and Cluster_21217.8 (NF-YA) were prioritized as candidate hubs, while Cluster_31326.1 (bZIP) and Cluster_11436.0 (C2H2) exhibited exclusively negative correlations with pathway genes, a pattern consistent with previously reported negative regulators in other species.

The current work is a discovery-phase candidate-screening study; all identified transcription factors and gene modules are based on co-expression correlations and have not been experimentally validated. Therefore, they should be regarded as candidate targets for future functional verification rather than confirmed regulators. The comprehensive multi-omics data, candidate co-expression modules, and prioritized transcription factors provided in this study offer a valuable resource and a clear framework for future investigation on the biosynthesis and regulation of active compounds in *P. corylifolia*.

## Data Availability

The raw RNA-seq data generated in this study have been deposited in the NCBI Sequence Read Archive (SRA) under BioProject accession number PRJNA1459288. The data are currently under temporary private access; reviewers can access the data via the following link: https://dataview.ncbi.nlm.nih.gov/object/SRR38419671?reviewer=dode9f6vm4j6os4aen0457dbq8 (accessed on 1 September 2026). The complete list of identified metabolites with Q1, Q3, DP, CE, confidence levels, and sample-level peak areas is provided in Supplementary Table S3. Owing to the proprietary nature of the MWDB database, retention times and matching scores for individual metabolites could not be released by the service provider. The R scripts used for all bioinformatic analyses are provided as annotated Supplementary Files (Files S1–S3).

## Supplementary Materials

The following supporting information can be downloaded at: https://www.mdpi.com/article/10.3390/biology15171567/s1, Supplementary Table S1. Information on reagents and the internal standard. Supplementary Table S2. Information on analytical instruments. Supplementary Table S3. List of all identified metabolites in *P. corylifolia*. Supplementary Table S4. Summary of OPLS-DA model diagnostic parameters for all pairwise tissue comparisons of *P. corylifolia*. Supplementary Table S5. Scale-free topology model fitting indices under different soft-thresholding powers. Supplementary Table S6. FDR-adjusted *p*-values for TF–pathway gene co-expression associations. All *p*-values shown are adjusted using the Benjamini–Hochberg procedure. Supplementary Table S7. Total metabolite proportion in *P. corylifolia*. Supplementary Table S8. Complete list of the 655 common differentially accumulated metabolites identified across the four pairwise tissue comparisons (root vs. fruit, stem vs. fruit, leaf vs. fruit, flower vs. fruit). Supplementary Table S9. KEGG pathway enrichment analysis of the 98 fruit-enriched DAMs with KEGG annotations. Supplementary Table S10. Metabolite enrichment analysis of the 98 fruit-enriched DAMs with available annotations. Supplementary Table S11. List of the 80 unique metabolites selected for tissue-specific accumulation heatmap visualization, with their chemical classes, tissue peak areas, whole-plant mean abundance, QC RSD values, and literature-reported status. Supplementary Table S12. Quality control of transcriptomic data in 15 samples. Supplementary Table S13. Read mapping statistics of all 15 transcriptome samples from *P. corylifolia*. Supplementary Table S14. Functional annotation statistics of *P. corylifolia* Unigenes against public databases. Supplementary Table S15. Differentially expressed genes (DEGs) identified across the four tissue comparisons versus fruits, with FPKM values, log_2_ fold changes, and adjusted *p*-values. Supplementary Table S16. Gene Ontology (GO) enrichment analysis results for the 4311 common differentially expressed genes. Supplementary Table S17. Transcription factors in *P. corylifolia*. Supplementary Table S18. Differentially expressed transcription factors (DETFs) identified in the four comparisons against fruits, with fold changes, *p*-values, adjusted *p*-values, and regulation status. Supplementary Table S19. Pearson correlation coefficients between selected pathway genes and corresponding metabolites across the 15 tissue samples. Supplementary Table S20. Detailed annotation information of putative enzyme genes involved in flavonoid and coumarin biosynthetic pathways. Supplementary Table S21. FPKM expression values of all putative enzyme genes in Figure 4 across five tissues. Supplementary Table S22. Relative peak area of all corresponding metabolites in Figure 4 across five tissues. Supplementary Table S23. TF–pathway gene co-expression associations with FDR-adjusted *p*-values. Supplementary Table S24. Core transcription factors identified in the magenta module. Supplementary Figure S1. Permutation test validation plots of representative OPLS-DA models from tissue pairwise comparisons in *P. corylifolia*. Supplementary Figure S2. BUSCO completeness assessment of the de novo transcriptome assembly of *P. corylifolia*. This bar plot summarizes the Universal Single-Copy Orthologs (BUSCO) evaluation results of the assembled unigenes. A total of 255 conserved single-copy ortholog groups were used as the reference dataset. The results include 253 complete BUSCOs (99.22%), consisting of 18 single-copy and 235 duplicated orthologs, 2 fragmented BUSCOs (0.78%), and 0 missing BUSCOs (0.00%), demonstrating high integrity and quality of the de novo transcriptome assembly. Supplementary Figure S3. Scale-free topology check at power = 26. Left: histogram of soft connectivity distribution; Right: linear regression of log10(k) against log10(p(k)) with R^2^ = 0.79 and slope = −0.9, confirming valid scale-free network construction. Supplementary Figure S4. (A) Total ion current chromatogram (TIC) of the quality control (QC) sample. (B) Coefficient of variation (CV) distribution (ECDF) plot for all metabolites. (C) Pearson correlation heatmap of the quality control (QC) sample. N, negative ion mode; P, positive ion mode. Supplementary Figure S5. Heatmap of tissue-specific correlations in metabolomic samples. This heatmap shows sample clustering based on Pearson correlation coefficients, with samples grouped by root, stem, leaf, flower, and fruit. Correlations between biological replicates within the same tissue exceed 0.98, indicating good biological reproducibility. Supplementary Figure S6. Pie chart showing the number proportion of all annotated metabolites across chemical classes. Supplementary Figure S7. Venn diagram of downregulated differential metabolites. Supplementary Figure S8. Metabolite enrichment analysis of differential metabolites. Supplementary Figure S9. (A) Principal Component Analysis (PCA) of the transcriptome. (B) Correlation analysis of transcriptome samples from different regions. Pearson’s correlation coefficient (r) was used as an indicator of correlation between biological replicates. The closer the absolute value of r is to 1 (the redder the color), the stronger the correlation between the two replicate samples. Supplementary Figure S10. Pie chart showing the classification of differentially expressed transcription factors (DETFs) across different parts of *Psoralea corylifolia* compared to the fruit. The figure illustrates the relative distribution of differentially expressed transcription factors (DETFs) across different families in four comparison groups: root vs. fruit (R vs. Fr), stem vs. fruit (S vs. Fr), leaf vs. fruit (L vs. Fr), and flower vs. fruit (Fl vs. Fr). Supplementary File S1. R script for metabolite accumulation heatmaps (Figure 2D–H). Supplementary File S2. R script for WGCNA network construction and Kwithin/Ktotal calculation (Figure 5D, Table S14). Supplementary File S3. R script for module–metabolite–trait correlation heatmap (Figure 5C).

## Abbreviations

The following abbreviations are used in this manuscript:

OH	Hydroxy
Me	methyl
Glc	glucoside
di-OH	dihydroxy
di-OH	dimethoxy
Mal	malonyl
Api	apiosyl
PCW organization/biogenesis	plant-type cell wall organization or biogenesis
O-glycosyl hydrolase activity	hydrolase activity, hydrolyzing O-glycosyl compounds
Phosphate transmembrane transporter activity	acyltransferase activity, transferring groups other than amino-acyl groups
Var. plant sec. metab. biosynthesis	Biosynthesis of various plant secondary metabolites
Ala, Asp & Glu metab.	Alanine, aspartate and glutamate metabolism
SDG biosynthesis	Stilbenoid, diarylheptanoid and gingerol biosynthesis
TPP alkaloid biosynthesis	Tropane, piperidine and pyridine alkaloid biosynthesis
DHK	Dihydrokaempferol
Nar chalcone	Naringenin chalcone

## Appendix A

### Full UPLC-MS/MS Instrument Acquisition Parameters

Complete instrument parameter settings for UPLC-MS/MS detection are provided in this appendix to ensure full experimental reproducibility. Simplified core parameters have been presented in Section 2.3.2 of the main text. The ultra-high performance liquid chromatography system used was ExionLC™ AD (SCIEX, Marlborough, MA, USA), coupled with a triple quadrupole mass spectrometer fitted with an electrospray ionization (ESI) source. Detailed ESI operating parameters are summarized in Table A1.

**Table A1:** Detailed ESI source parameters for mass spectrometry detection.

**Parameter**	**Value**
Ion source temperature	500 °C
IonSpray voltage	5500 V (positive ion mode), −4500 V (negative ion mode)
Nebulizer gas (GS1)	50 psi
Auxiliary gas (GS2)	60 psi
Curtain gas (CUR)	25 psi
Collision-induced dissociation level	High
Collision gas (nitrogen)	Medium
Mass tolerance	20 ppm
Acquisition mode	Multiple reaction monitoring (MRM)
