# Supplementary material for: Integrated Transcriptomic and Metabolomic Analyses Identify Candidate Transcription Factors Associated with Flavonoid and Coumarin Accumulation in Psoralea corylifolia
Source: Biology (Basel). 2026 Sep 7;15(17):1567. doi: 10.3390/biology15171567 (PMC13565213; doi:10.3390/biology15171567)
Supplement: Supplementary file 1 [file biology-15-01567-s001.zip › Supplementary File Figures.pdf]

Supporting Information

The following supplemental materials is available for this article:

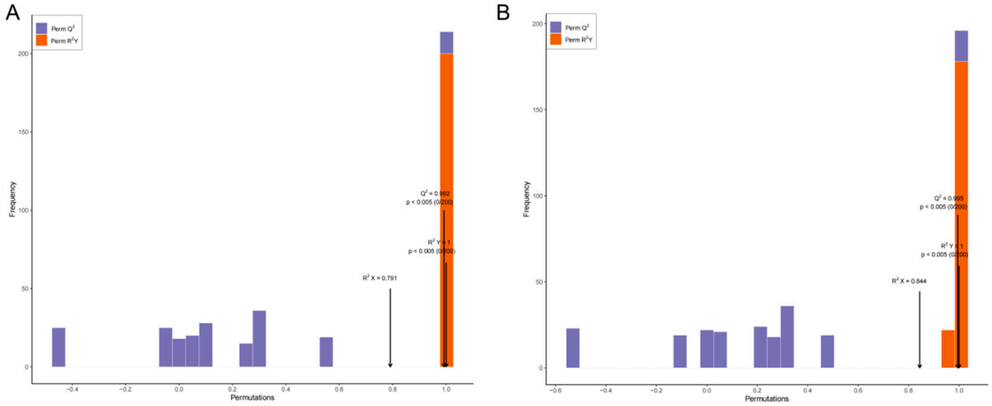

Supplementary Figure S1. Permutation test validation plots of representative OPLS-DA models from tissue pairwise comparisons in *P. corylifolia.*

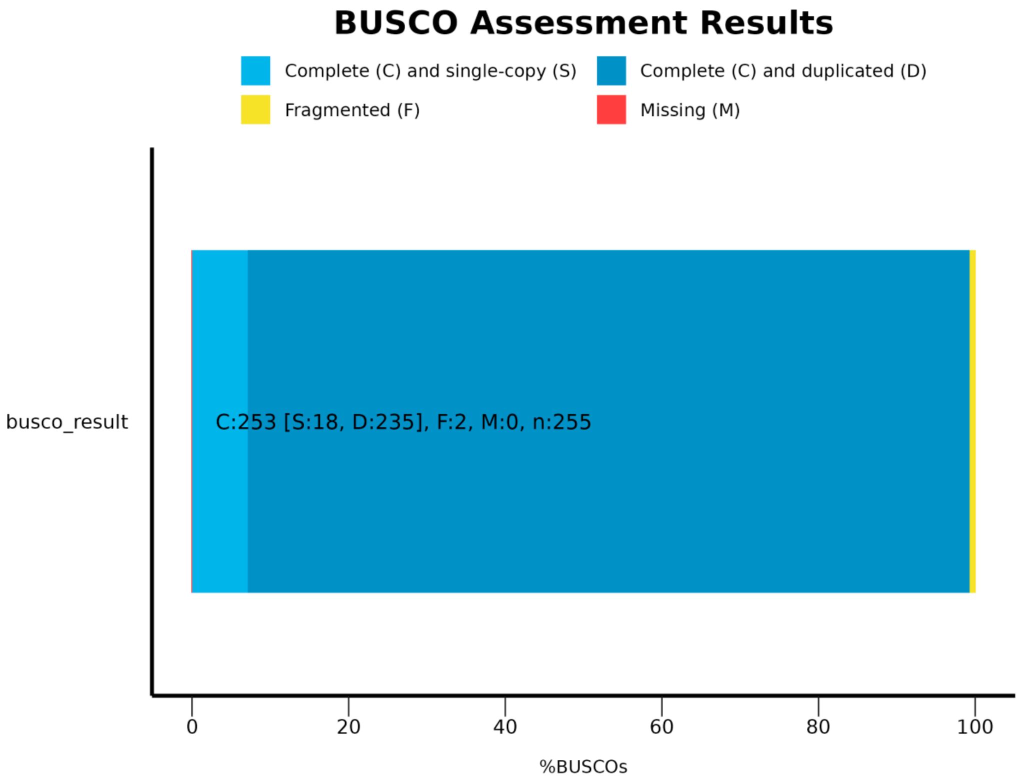

Supplementary Figure S2. BUSCO completeness assessment of the de novo transcriptome assembly of *P. corylifolia.* This bar plot summarizes the Benchmarking Universal Single-Copy Orthologs (BUSCO) evaluation results of the assembled unigenes. A total of 255 conserved single-copy ortholog groups were used as the reference dataset. The results include 253 complete BUSCOs (99.22%), consisting of 18 single-copy and 235 duplicated orthologs, 2 fragmented BUSCOs (0.78%), and 0 missing BUSCOs (0.00%), demonstrating high integrity and quality of the de novo transcriptome assembly.

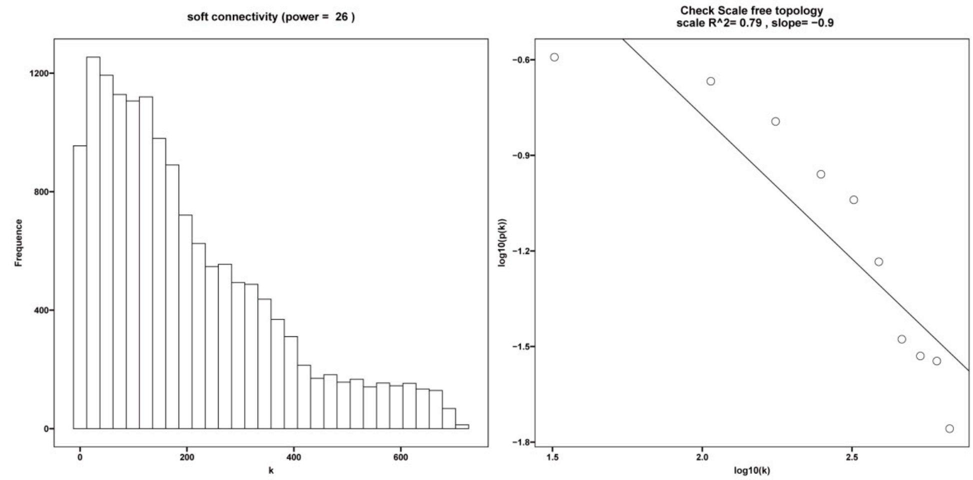

Supplementary Figure S3. Scale-free topology check at power = 26. Left: histogram of soft connectivity distribution; Right: linear regression of $\log_{10}$ (k) against log10 (p (k)) with R^2=0.79 and slope -0.9, confirming valid scale-free network construction.

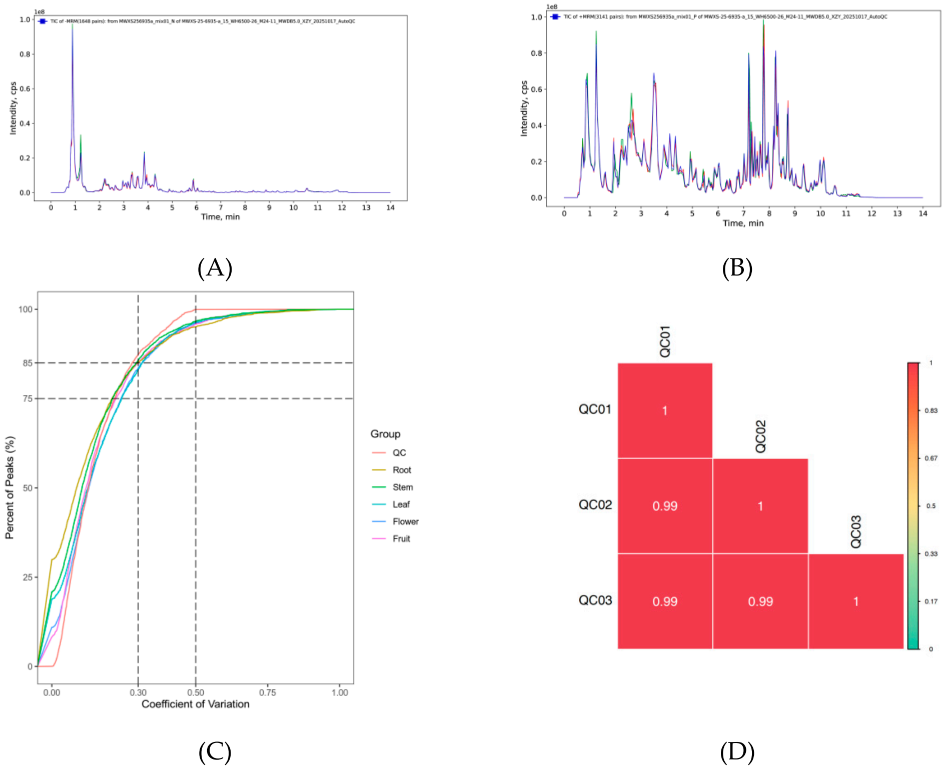

Supplementary Figure S4. (A) Total ion current chromatogram (TIC) of the quality control (QC) sample. (B) Coefficient of variation (CV) distribution (ECDF) plot for all metabolites. (C) Pearson correlation heatmap of the quality control (QC) sample. N: Negative ion mode; P: Positive ion mode.

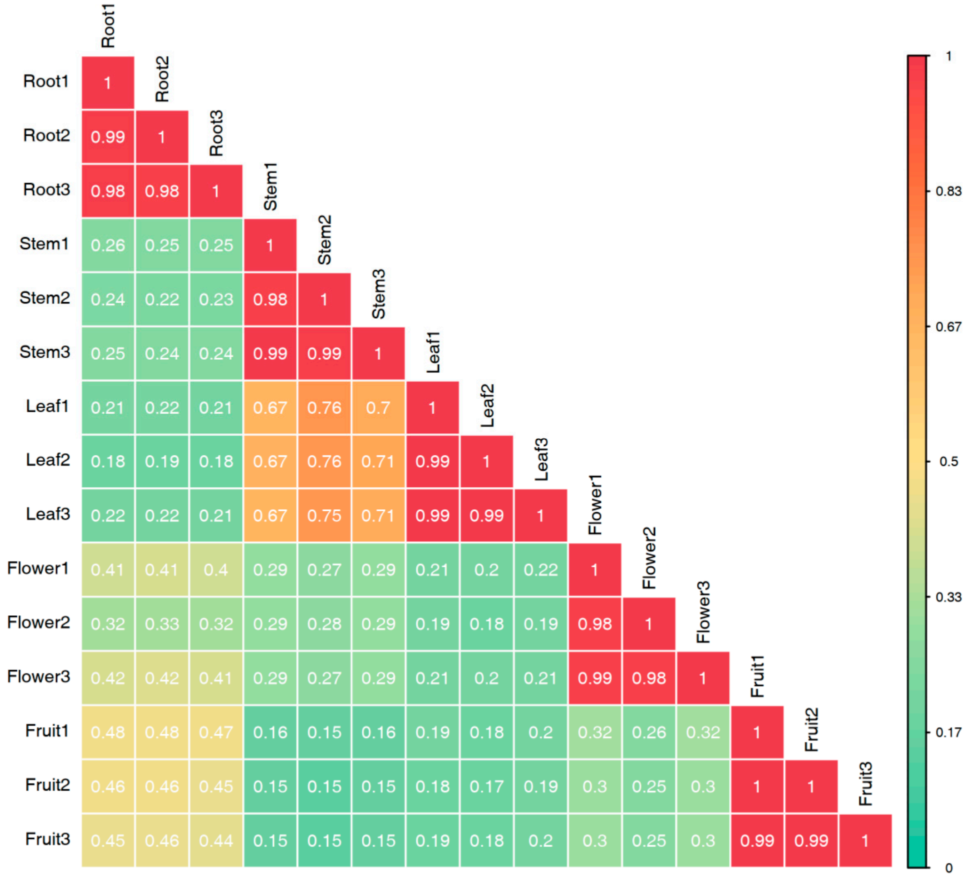

Supplementary Figure S5. Heatmap of tissue-specific correlations in metabolomic samples. This heatmap shows sample clustering based on Pearson correlation coefficients, with samples grouped by root, stem, leaf, flower, and fruit. Correlations between biological replicates within the same tissue exceed 0.98, indicating good biological reproducibility.

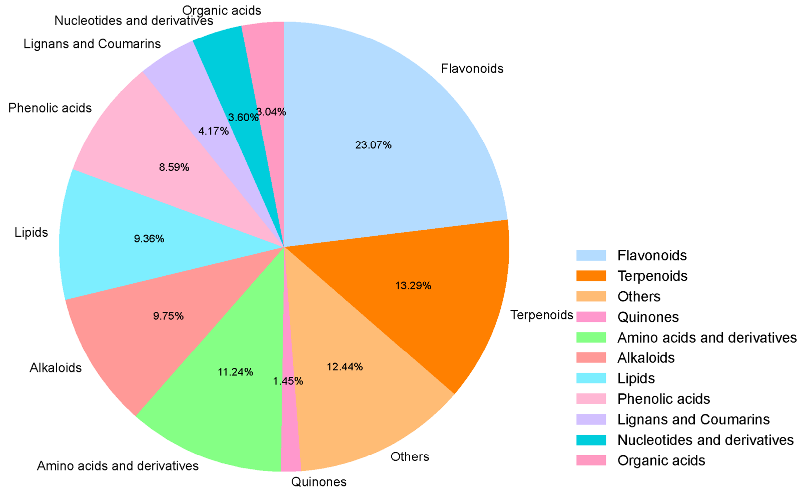

Supplementary Figure S6. Pie chart showing the number proportion of all annotated metabolites across chemical classes.

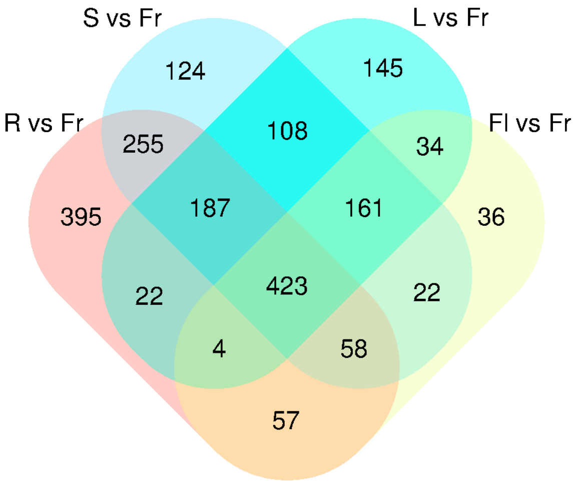

Supplementary Figure S7. Venn diagram of downregulated differential metabolites.

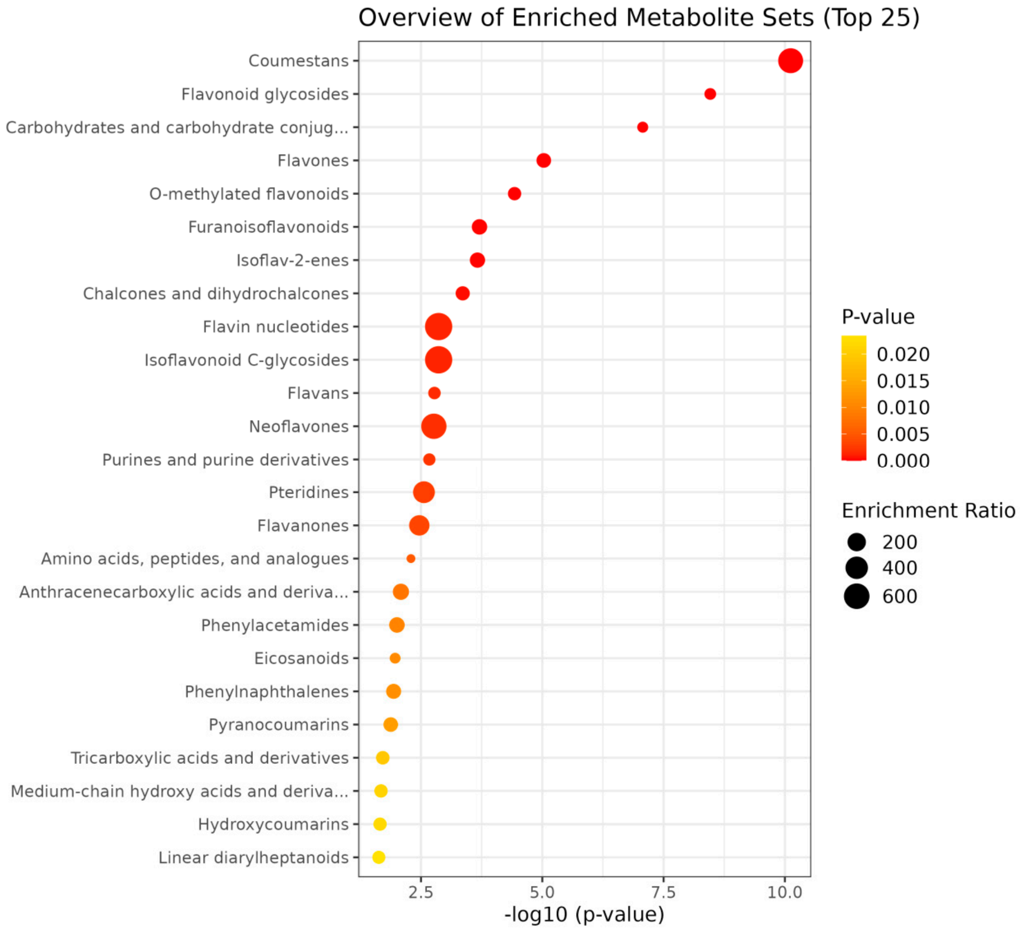

Supplementary Figure S8. Metabolite enrichment analysis of differential metabolites.

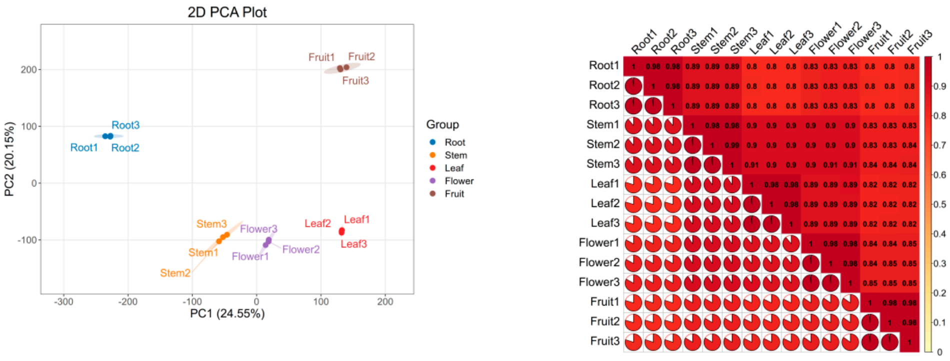

(A) (B)

Supplementary Figure S9. (A) Principal Component Analysis (PCA) of the transcriptome. (B) Correlation analysis of transcriptome samples from different regions. Pearson's correlation coefficient (r) was used as an indicator of correlation between biological replicates. The closer the absolute value of r is to 1 (the redder the color), the stronger the correlation between the two replicate samples.

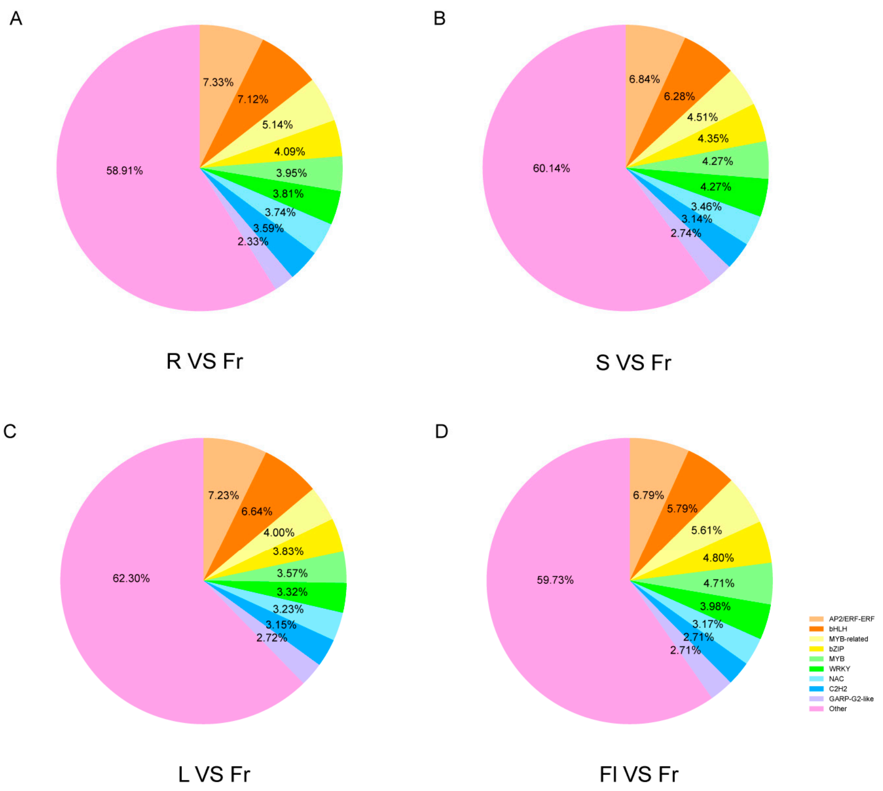

Supplementary Figure S10. Pie chart showing the classification of differentially expressed transcription factors (DETFs) across different parts of Psoralea corylifolia compared to the fruit.The figure illustrates the relative distribution of differentially expressed transcription factors (DETFs) across different families in four comparison groups: root vs. fruit (R vs. Fr), stem vs. fruit (S vs. Fr), leaf vs. fruit (L vs. Fr), and flower vs. fruit (Fl vs. Fr).
